# Two novel blue phosphorescent host materials containing phenothiazine-5,5-dioxide structure derivatives

**DOI:** 10.3762/bjoc.14.73

**Published:** 2018-04-17

**Authors:** Feng-Ming Xie, Qingdong Ou, Qiang Zhang, Jiang-Kun Zhang, Guo-Liang Dai, Xin Zhao, Huai-Xin Wei

**Affiliations:** 1 College of Chemistry, Biology and Material Engineering, Suzhou University of Science and Technology, Suzhou, Jiangsu 215009, P.R. China; 2 Jiangsu Key Laboratory of Environmental Functional Materials, Suzhou, Jiangsu 215009, P.R. China; 3 Department of Materials Science and Engineering, Monash University, Clayton, Victoria 3800, Australia

**Keywords:** host materials, phenothiazine, phosphorescence, synthesis

## Abstract

Two novel D–A bipolar blue phosphorescent host materials based on phenothiazine-5,5-dioxide: 3-(9*H*-carbazol-9-yl)-10-ethyl-10*H*-phenothiazine-5,5-dioxide (CEPDO) and 10-butyl-3-(9*H*-carbazol-9-yl)-10*H*-phenothiazine-5,5-dioxide (CBPDO) were synthesized and characterized. The photophysical, electrochemical and thermal properties were systematically investigated. CEPDO and CBPDO not only have a high triplet energy but also show a bipolar behavior. Moreover, their fluorescence emission peaks are in the blue fluorescence region at 408 nm and the fluorescence quantum efficiency (Φ) of CEPDO and CBPDO were 62.5% and 59.7%, respectively. Both CEPDO and CBPDO showed very high thermal stability with decomposition temperatures (*T*_d_) of 409 and 396 °C as well as suitable HOMO and LUMO energy levels. This preferable performance suggests that CEPDO and CBPDO are alternative bipolar host materials for the PhOLEDs.

## Introduction

Since 1987, the Tang group [1] firstly reported double organic light-emitting diodes (OLEDs) with ultra-thin film by using vacuum evaporation technology. It has attracted much attention to develop it. In recent years, OLEDs have been rapidly developed and widely used in lighting and display because of many unique performance advantages such as wide viewing angle (≥170°), fast response (1 μs scale), high luminous efficiency, low drive voltage (3–10 V), thickness (less than 2 mm), lightweightness and flexibility [2–6]. Compared with traditional fluorescence OLEDs which only utilize singlet (25%) excitons for electroluminescence, PhOLEDs can simultaneously harvest both the singlet and triplet (75%) excitons through spin-orbit coupling (SOC), and obtain nearly 100% of the internal quantum efficiency (IQE). Thus, most of researchers have focused on phosphorescent organic light-emitting diodes (PhOLEDs) all over the world [7–8]. With the deepening of this research, the performance of red and green electroluminescent devices has been able to meet the commercial requirements, but the blue electroluminescent devices have several weaknesses such as low efficiency and poor stability and so on, which hinder its development. It has been proved that the selection of proper materials for each layer is very important for achieving highly efficient PhOLEDs. In particular, the design of host materials plays a critical role in the determination of the devices performance. Therefore, it is beneficial to develop new blue phosphorescent host materials with high-performance for blue PhOLEDs [9–12].

Generally, ideal host materials are required to fulfill several requirements [13–14]: i) the triplet energy level (*E*_T_) should be higher for efficient energy transfer to the guest; ii) suitable energy levels appropriately aligned with those of the neighboring active layers for efficient charge carrier injection to achieve a low operating voltage; iii) good and balanced charge carrier transport properties for the hole–electron recombination process; iv) good thermal and morphological stability for the vacuum deposition method to prolong the device operational lifetime.

Carbazole groups are widely used in host materials because of their high triplet energy levels and high hole mobility [15]. The Lee group [16] linked carbazolyl groups to diphenyl phosphoramines to design asymmetric (9-phenyl-9*H*-carbazole-2,5-diyl)bis(diphenylphosphine oxide) (PCPOs) with a higher triplet energy level (2.80 eV) and a glass transition temperature (140 °C). The maximum external quantum efficiency (EQE) of PhOLEDs was 31.4%, which was prepared by PCPO as a bipolar host material. Kim et al. [17] reported that the bipolar host material 9-(4-(9*H*-pyrido[2,3-*b*]indol-9-yl)phenyl)-9*H-*3,9'-bicarbazole (pBCb2Cz) has a high triplet energy level (2.93 eV), which is the main material of blue PhOLEDs, and the EQE of the device is 23.0%. The Suh group [18] reported that the EQE of the prepared device of the bipolar host material *N*-(3,5-di(9*H*-carbazol-9-yl)phenyl)-*N*-(pyridin-2-yl)pyridin-2-amine (DCPPy) based on carbazole group is 21.6%. The Wang group [19] designed a host material with symmetrical structure based on phenothiazine-5,5-dioxide. But the host materials with asymmetric structure based on the phenothiazine-5,5-dioxide were rarely reported. For obtaining a high triplet energy level and good stability, herein, with phenothiazine-5,5-dioxide as acceptor (A) and carbazole as donor (D), and introducing an alkane chain group to the host materials for better film-forming properties, two novel blue phosphorescent host materials, CEPDO and CBPDO, were synthesized. At the same time, the photophysical properties, electrochemical properties and their thermal stability were studied and the expected results were obtained.

## Results and Discussion

### Synthesis and theoretical calculations

**Scheme 1:**
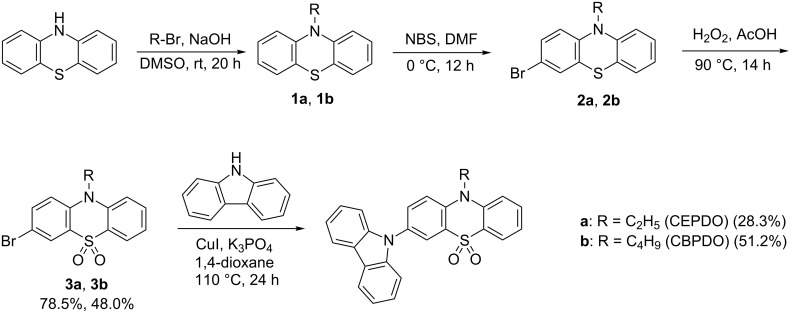
Synthetic routes of CEPDO and CBPDO.

The synthesis route for CEPDO and CBPDO is shown in Scheme 1. The detailed synthesis procedures and characterizations are given in Supporting Information File 1.

**Figure 1:**
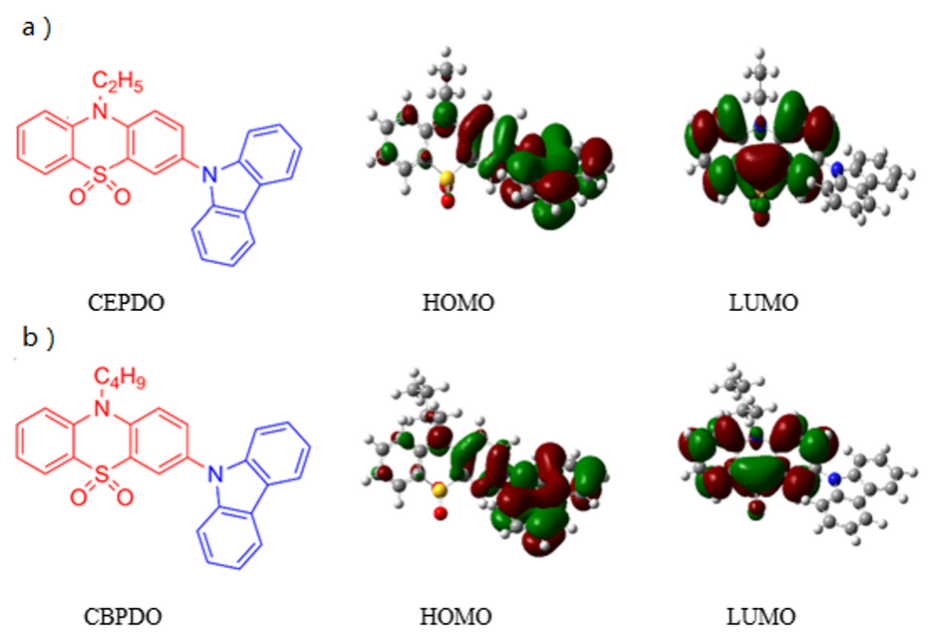
Structures and molecular orbitals of (a) CEPDO and (b) CBPDO.

In order to further understand the structural properties of the materials and the possibility of charge transfer from donor to acceptor on electronic excitation, the electronic structure of the materials were analyzed by density functional theoretical (DFT) calculations using the Gaussian 09 program package. The electron density distributions and energy levels of the HOMO and LUMO are displayed in Figure 1.

The HOMOs of CEPDO and CBPDO are mainly distributed over the electron-donating carbazole moiety and slightly extended to the phenyl ring. The LUMOs are mostly localized on the phenothiazine-5,5-dioxide, based on the DFT calculation. There is a small degree of spatial overlap between the HOMO and LUMO in these two molecules. The separated HOMO and LUMO resulted from the strong electron-donating nature of the carbazole unit and electron-withdrawing ability of the phenothiazine-5,5-dioxide unit, thus realized the orbital separation of hole and electron transport in the same molecule. This indicated CEPDO and CBPDO have bipolar characteristic.

### Photophysical properties

**Figure 2:**
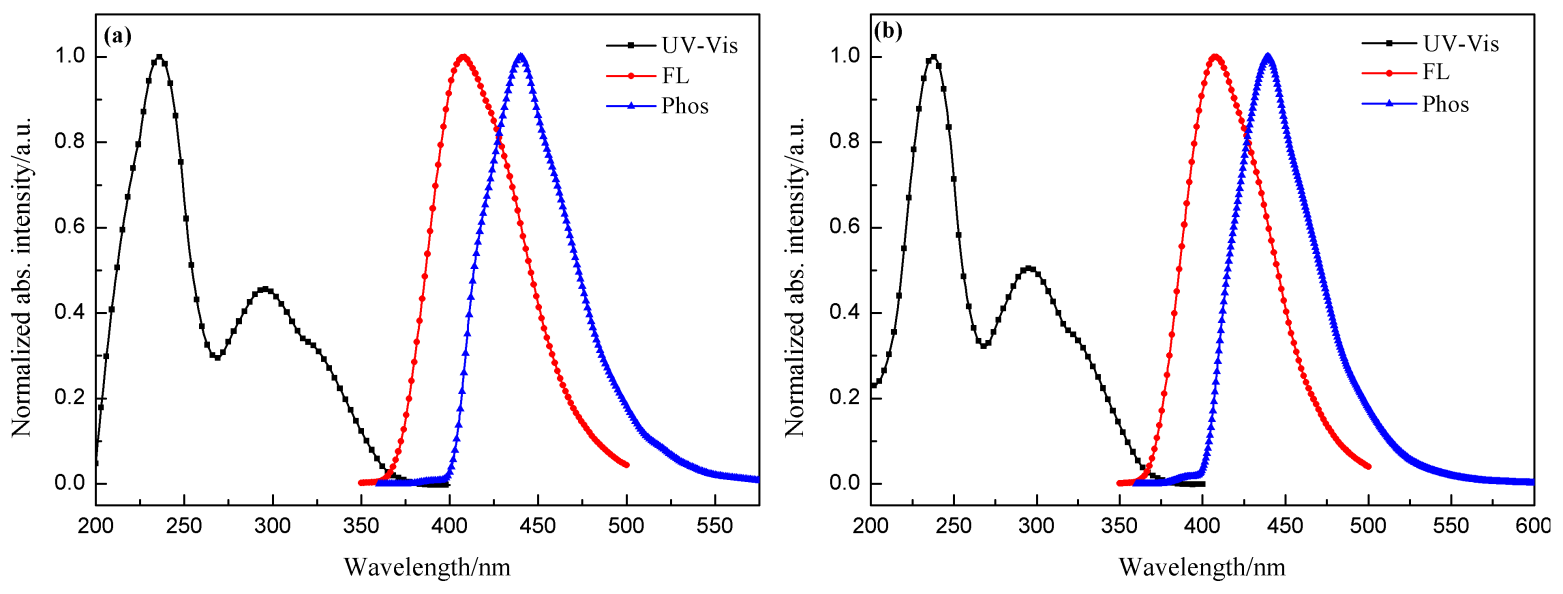
UV–vis absorption and photoluminescence spectra in DCM solution (1 × 10^−5^ M and 1 × 10^−6^ M, respectively) at room temperature, phosphorescence spectra in 2-MeTHF solution (1 × 10^−3^ M) at 77 K of CEPDO (a) and CBPDO (b).

Figure 2 presents the UV–vis absorption, photoluminescence and phosphorescence (77 K) spectra of CEPDO (a) and CBPDO (b) in solution, respectively. Obviously, the strong absorption peak at 236 nm can be ascribed to the π→π* transition of carbazole moiety of the molecules, and the weaker absorptions around 295 nm assign to the n→π* transition of the conjugation of the whole molecule.

The optical bandgap (*E*_g_) of the two substances were all calculated to be 3.32 eV from the UV–vis absorption spectra of CEPDO and CBPDO. Upon photoexcitation of 330 nm at room temperature, both CEPDO and CBPDO exhibited a FL spectrum with peaks at 408 nm and emitted blue fluorescence. The fluorescence quantum efficiencies (Φ) of CEPDO and CBPDO were 62.5% and 59.7%, respectively, by using quinine sulfate as a reference [20]. Compared with CEPDO, the longer alkyl chain of CBPDO led to a corresponding increase in Φ value. Therefore, CEPDO and CBPDO are promising photoelectric materials. To obtain the triplet energy level, their low-temperature Phos spectra were measured in a 2-methyltetrahydrofuran solution at 77 K which are also shown in Figure 2. The phosphorescence emission peaks were at 440 nm and 439 nm, respectively. According to the onset of their phosphorescence spectra, the calculated triplet energy levels (*E*_T_) of CEPDO and CBPDO were identical at 2.82 eV, which matched with the blue phosphorescent guest material FIrpic (2.65 eV), dark blue phosphorescent guest material FCNIrpic (2.74 eV) and FIr6 (2.73 eV). The high *E*_T_ was attributed to the insulated carbazole moieties. Hence, both CEPDO and CBPDO are expected to be applied to PhOLED as a blue phosphorescent host material.

### Electrochemical properties

**Figure 3:**
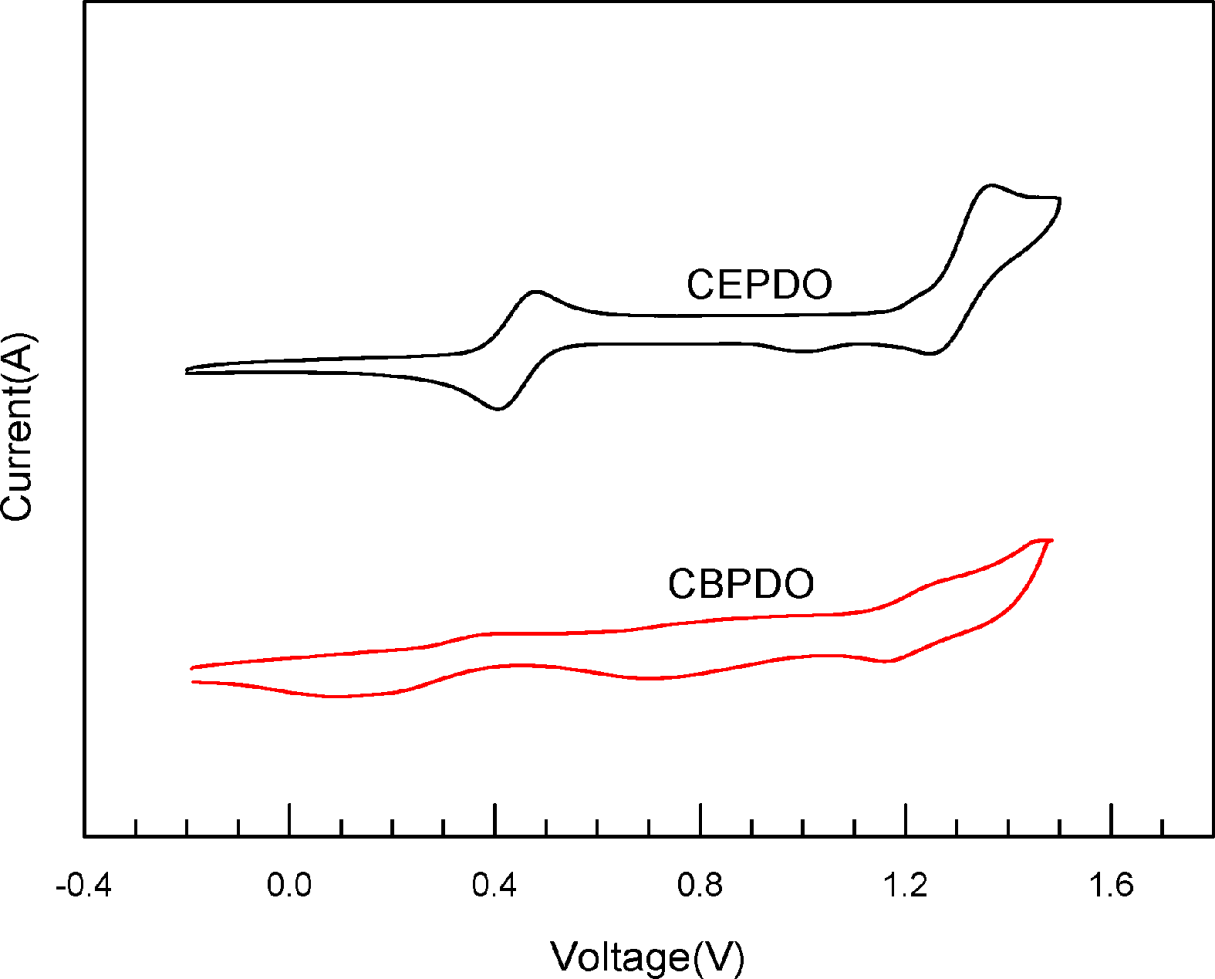
Cyclic voltammograms for CEPDO and CBPDO in DCM solution.

The electrochemical properties of CEPDO and CBPDO were studied by cyclic voltammetry (CV) measurements in deoxygenated DCM solution with 0.1 M tetrabutylammonium hexafluorophosphate as the supporting electrolyte. The cyclic voltammograms are shown in Figure 3.

Cyclic voltammetry was measured with a glassy carbon working electrode, a platinum wire counter electrode, a saturated Ag/AgCl reference electrode, ferrocenium-ferrocene (Fc^+^/Fc) as the internal standard and tetrabutylammonium hexafluorophosphate (0.1 M) as the supporting electrolyte.

The onset potential (*E*^onset^_ox_) of the first oxidation wave for CEPDO and CBPDO are utilized to estimate the HOMO energy level according to the equation *E*_HOMO_*= −e(E*^onset^_ox_* +* 4.8*)* as ca. −5.63 and −5.64 eV, respectively. And both HOMO energy levels are matched with the functional function of the anode ITO (−4.5 to −5.0 eV). The LUMO energy levels of CEPDO and CBPDO are estimated from the half-potential to be −2.31 eV and −2.32 eV, respectively, which are matched with the LUMO energy level of electron injection material TAZ and favorable for electron injection and transmission [21]. Therefore, we successfully synthesized two novel bipolar host materials with higher triplet energy by choosing suitable donor and acceptor units.

### Thermal properties

**Figure 4:**
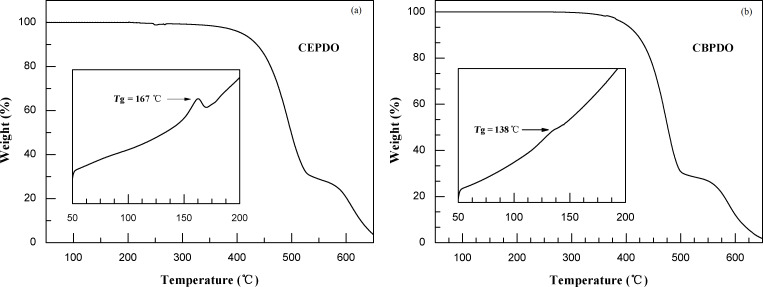
DSC and TGA curves of CEPDO and CBPDO.

The thermal properties of CEPDO and CBPDO were determined by thermogravimetric analysis (TGA) and differential scanning calorimetry (DSC) under nitrogen atmosphere at a scanning rate of 10 °C/min and the results are shown in Figure 4. Both CEPDO and CBPDO show very high thermal stability with decomposition temperatures (*T*_d_) of 409 and 396 °C and glass transition temperatures (*T*_g_) of 167 and 138 °C, respectively. Compared to the introduction of ethyl groups, the introduction of normal butyl groups to the phenothiazine-5,5-dioxide moieties appears to decrease the *T*_d_ and *T*_g_ of CEPDO by 13 °C and 29 °C, respectively, relative to those of CBPDO. The reason may be the *n*-butyl chain is longer. It reduces the polarity and intermolecular forces of molecules. The high thermal values ensure high thermostability and that the amorphous structure can form homogeneous and stable films by vacuum deposition to improve the lifetime of the PhOLEDs. The photophysics, electrochemical and thermal properties of CEPDO and CBPDO are summarized in Table 1.

**Table 1:** Photophysics, electrochemical and thermal properties of CEPDO and CBPDO.

compound	λ_abs_^a^ /nm	λ_onset_^b^ /nm	λ_emt_^c^ /nm	λ_phos_^d^ /nm	Φ^e^ /%	*E*_onset_^f^ /eV	*E*_g_^g^ /eV	*E*_t_^h^ /eV	*E*_HOMO/LUMO_^i^ /eV	*T*_d/g_^j^ /°C

CEPDO	236	373	408	440	49	1.23	3.32	2.82	−5.63/−2.31	409/167
CBPDO	236	372	408	439	69	1.24	3.32	2.82	−5.64/−2.32	396/138

^a^The absorption maximum of the UV–vis spectrum; ^b^estimated from the onset of the UV–vis spectrum; ^c^emission fluorescence maximum at room temperature; ^d^phosphorescence emission peak at 77 K; ^e^fluorescence quantum yield; ^f^first oxidation peak potential; ^g^*E*_g_ = 1240/*λ*_onset_; ^h^*E*_T_ = 1240/*λ*_phos_; ^i^*E*_HOMO_: measured from the oxidation potential in 10^−3^ M DCM solution by cyclic voltammetry, *E*_LUMO_ = *E*_g_ + *E*_HOMO_; ^j^decomposition temperature (*T*_d_) with 5% loss, glass transition temperature (*T*_g_).

## Conclusion

In summary, we have designed and synthesized two bipolar host materials CEPDO and CBPDO. CEPDO and CBPDO not only have a high triplet energy but also show a bipolar behavior. Moreover, their fluorescence emission peaks are blue fluorescence at 408 nm and the fluorescence quantum efficiency (Φ) of CEPDO and CBPDO are 62.5% and 59.7%, respectively. Both CEPDO and CBPDO show very high thermal stability with *T*_d_ of 409 and 396 °C, *T*_g_ of 167 and 138 °C, respectively, and also appear suitable HOMO and LUMO energy levels. Hence, CEPDO and CBPDO are two promising blue phosphorescent host materials for PhOLEDs.

## Supporting Information

File 1 Experimental part and copies of NMR spectra.

## References

[1] Tang C W, VanSlyke S A (1987). Appl Phys Lett.

[2] Rajamalli P, Senthilkumar N, Gandeepan P, Huang P-Y, Huang M-J, Ren-Wu C-Z, Yang C-Y, Chiu M-J, Chu L-K, Lin H-W (2016). J Am Chem Soc.

[3] Baldo M A, O'Brien D F, You Y, Shoustikov A, Sibley S, Thompson M E, Forrest S R (1998). Nature.

[4] Lin T, Zhang T, Song Q, Jin F, Liu Z, Su Z, Luo Y, Chu B, Lee C S, Li W (2016). Org Electron.

[5] Kim K-H, Yoo S-J, Kim J-J (2016). Chem Mater.

[6] Feng M Q, Tang J X, Liao L S (2017). Imaging Sci Photochem.

[7] Dos Santos P L, Ward J S, Bryce M R, Monkman A P (2016). J Phys Chem Lett.

[8] Wang F, Tao Y, Huang W (2015). Acta Chim Sin.

[9] Xiao L, Chen Z, Qu B, Luo J, Kong S, Gong Q, Kido J (2011). Adv Mater.

[10] Seino Y, Sasabe H, Pu Y-J, Kido J (2014). Adv Mater.

[11] Tao Y, Yang C, Qin J (2011). Chem Soc Rev.

[12] Chaskar A, Chen H-F, Wong K-T (2011). Adv Mater.

[13] Zhao Z, Yu G, Chang Q, Liu X, Liu Y, Wang L, Liu Z, Bian Z, Liu W, Huang C (2017). J Mater Chem C.

[14] Sun Q, Cui L-S, Xie Y-M, Liang J-J, Jiang Z-q, Liao L-s, Fung M-K (2017). Org Electron.

[15] Baryshnikov G V, Bondarchuk S V, Minaeva V A, Ågren H, Minaev B F (2017). J Mol Model.

[16] Kim M, Lee J Y (2014). Adv Funct Mater.

[17] Kim S J, Kim Y J, Son Y H, Hur J A, Um H A, Shin J, Lee T W, Cho M J, Kim J K, Joo S (2013). Chem Commun.

[18] Park S-R, Kim S-M, Kang J-H, Lee J-H, Suh M C (2017). Dyes Pigm.

[19] Zheng Y, Xie Q, Wang B (2015). Chin J Org Chem.

[20] Yang X, Pan Z T, Ma Y J (2003). Anal Sci.

[21] Li J, Zhang Z, Han C, Ding D, Zhao Y, Huang W, Xu H (2015). J Mater Chem C.

