# Supplementary material for: Two novel blue phosphorescent host materials containing phenothiazine-5,5-dioxide structure derivatives
Source: Beilstein J Org Chem. 2018 Apr 17;14:869–74. doi: 10.3762/bjoc.14.73 (PMC5942372; doi:10.3762/bjoc.14.73)
Supplement: File 1 — Experimental part and copies of NMR spectra. [file Beilstein_J_Org_Chem-14-869-s001.pdf]

Supporting Information

for

Two novel blue phosphorescent host materials containing phenothiazine-5,5-dioxide structure derivatives

Feng-Ming Xie[1,2], Qingdong Ou[3], Qiang Zhang[1,2], Jiang-Kun Zhang[1,2], Guo-Liang Dai[1,2], Xin Zhao[1,2,*] and Huai-Xin Wei[1,2,*]

Address: [1]College of Chemistry, Biology and Material Engineering, Suzhou University of Science and Technology, Suzhou, Jiangsu, 215009,P.R. China, [2]Jiangsu Key Laboratory of Environmental Functional Materials, Suzhou, Jiangsu, 215009, P.R.China and [3]Department of Materials Science and Engineering, Monash University, Clayton, Victoria 3800, Australia

[1]Email: Xin Zhao - zhaoxinsz@126.com, Huaixin Wie - hxwei@usts.edu.cn

*Corresponding author

Experimental part and copies of NMR spectra

Materials and methods

Carbazole, bromoethane and bromobutane were purchased from Energy Chemical. Phenothiazine was purchased from Zhengzhou Alfachem Co., Ltd. The purity of these materials and other reagents are AR grade or higher. The moisture was removed by distillation before using the solvent. Part of the reaction was carried out under the protection of nitrogen. ^{1}H NMR and ^{13}C NMR spectra were recorded with a Bruker AVANCE Ⅲ type NMR Spectrometer (400 MHz) at room temperature. Melting points were measured using a Shanghai Precision Science Instrument Co., Ltd. SGW X-4 micro-melting point instrument. The UV–vis absorption spectra were recorded with a TU-1901 UV–vis spectrophotometer. The fluorescence luminescence spectra were measured using a LS55 type fluorescence spectrophotometer from PerkinElmer, USA. The low-temperature phosphorescence spectra were measured using a FLS 920 spectrometer manufactured by Einburgh Corporation and were measured in solution with 2-methyltetrahydrofuran as a solvent at 77 K. Cyclic voltammetry (CV) was measured on a RST 3100 electrochemical work station with a platinum carbon working electrode, a platinum wire counter electrode, a Ag/AgCl reference electrode, ferroceniume-ferrocene (Fc^{+}/Fc) as the internal standard and tetrabutylammonium hexafluorophosphate (0.1 M) as the supporting electrolyte. The cyclic voltammograms were obtained at a scanning rate of 20 mV s^{-1} with an argon-purged solution. TGA was conducted with a HCT-2 instrument at a heating rate of 10 °C $minute^{-1}$ in a nitrogen atmosphere. DSC were performed on a Pyris Diamond DSC Thermal Analyzer under a N_2 flow at a heating rate of 10 °C min^{-1}.

Synthesis of compound 1a. Phenothiazine (4.99 g，25 mmol) and NaOH (8.00 g，200.0 mmol) were added in dry DMSO (50 mL). Bromoethane (2.86 g，26.25 mmol) was added dropwise to this solution after stirring for 1 h under light shielding. The reaction mixture was stirred at room temperature for 20 h. The mixture was poured into water (200 mL) and extracted with CH_2Cl_2 (3 × 25 mL). The organic phase was dried over anhydrous $MgSO_4$, filtered and concentrated under reduced pressure. The crude product was purified by silica gel column chromatography (DCM/PE = 1:1) to give 4.87 g (85.8%) of **1a** as a yellow oil. ^{1}H NMR (400 MHz, $CDCl_3$): δ 7.24 - 7.05 (m, 4H), 7.03 - 6.77 (m, 4H), 3.95 (q, J = 7.0 Hz, 2H), 1.44 (t, J = 7.0 Hz, 3H); ^{13}C NMR (100 MHz, $CDCl_3$): δ 145.02, 127.32, 124.44, 122.33, 115.12, 41.74, 13.06.

Synthesis of compound 2a. A mixture of **1a** (3.00 g, 13.0 mmol) and DMF was cooled in an ice bath. After 20 minutes, NBS solution (2.82 g, in 20 mL DMF, 15.8 mmol) was added dropwise to this solution. The reaction mixture was stirred at room temperature for 12 h under light shielding. The mixture was poured into water (200 mL) and extracted with CH_2Cl_2 (3 × 25 mL). The organic phase was dried over anhydrous $MgSO_4$, filtered and concentrated under reduced pressure. The crude product was purified by silica gel column chromatography (DCM/PE = 1:1) to give 2.23 g (56.1%) of **2a** as a brown oil. ^{1}H NMR (400 MHz, $CDCl_3$)：δ 7.26 - 7.20 (m, 2H), 7.19 - 7.05 (m, 2H), 7.00 - 6.80 (m, 2H), 6.76 - 6.60 (m, 1H), 4.05 - 3.73 (m, 2H), 1.49 - 1.33 (m, 3H); ^{13}C NMR (100 MHz, $CDCl_3$)：δ 144.63, 144.19, 133.02, 131.91, 129.88, 129.55, 127.54, 122.67, 121.72, 116.16, 115.26, 41.89, 12.92.

Synthesis of compound 3a. H_2O_2 (25 mL) was added to a stirred solution of **2a** (3.06 g, 10.0 mmol) in glacial acetic acid (50 mL). The reaction mixture was refluxed at 85 °C for 24 h. The mixture was poured into water (200 mL) and extracted with ethyl acetate (3 × 25 mL). The

organic phase was dried over anhydrous $MgSO_4$, filtered and concentrated under reduced pressure. The crude product was purified by silica gel column chromatography (EA : PE = 2 : 1) to give 2.56 g (75.8%) of **3a** (m.p. 148.4 – 150.2 °C) as a white solid. ^1H NMR (400 MHz, $CDCl_3$): δ 8.22 (dd, J = 5.8, 2.4 Hz, 1H), 8.12 (dd, J = 7.9, 1.6 Hz, 1H), 7.75 - 7.58 (m, 2H), 7.38 (d, J = 8.6 Hz, 1H), 7.32 - 7.27 (m, 1H), 7.25 (s, 1H), 4.32 - 4.19 (m, 2H), 1.54 (dd, J = 14.1, 7.1 Hz, 3H); ^{13}C NMR (100 MHz, $CDCl_3$): δ 140.25, 139.44, 136.32, 136.03, 133.50, 126.23, 123.83, 122.15, 117.55, 115.73, 114.00, 43.35, 12.50.

Synthesis of CEPDO. K_3PO_4 (3.18 g, 15.0 mmol) and *trans*-1,2-cyclohexanediamine (0.11 g, 1.0 mmol) were added to a mixture of **3a** (1.00 g, 3.0 mmol) and carbazole (0.55 g, 3.3 mmol) in dry 1,4-dioxane (15 mL). Then CuI (0.19 g, 1.0 mmol) was added to this solution under the protection of N_2. The mixture was stirred at 110 °C for 36 h. After cooling, the mixture was poured into water (100 mL) and extracted with CH_2Cl_2 (2 × 20 mL). The organic phase was dried over anhydrous $MgSO_4$, filtered and concentrated under reduced pressure. The crude product was purified by silica gel column chromatography (DCM/PE = 1:1) to give 0.36 g (28.3%) of **CEPDO** (m.p. 223.0–224.5 °C) as a white solid. ^1H NMR (400 MHz, $CDCl_3$): δ 8.38 (dd, J = 16.9, 2.5 Hz, 1H), 8.16 (dd, J = 6.7, 4.8 Hz, 3H), 7.82 (dd, J = 9.0, 2.5 Hz, 1H), 7.75 - 7.65 (m, 1H),7.61 (d, J = 9.1 Hz, 1H), 7.51 - 7.37 (m, 5H), 7.39 - 7.28 (m, 3H), 4.40 (q, J = 7.1 Hz, 2H), 1.66 (t, J = 7.1 Hz, 3H); ^{13}C NMR(100 MHz，$CDCl_3$): δ 140.76, 140.40, 139.49, 139.30, 133.53, 132.55 -131.89, 131.71, 126.27, 125.05, 124.93, 123.86, 123.56, 122.20, 120.71 -120.26, 117.64), 117.43, 115.81, 109.48, 99.99, 43.51, 12.70. Anal. Calcd for $C_{26}H_{20}N_2O_2S$: C, 73.56; H, 4.75; N, 6.60; S, 7.55. Found: C, 73.52; H, 4.76; N, 6.58; S, 7.59.

Synthesis of 1b. A procedure similar to the synthesis of **1a.** The amount of the same substance C_2H_5Br was replaced by C_4H_9Br, and give 5.74 g (90.1%) of **1b** as brown oil. ^{1}H NMR (400 MHz, $CDCl_3$) : δ 7.27 - 7.07 (m, 4H), 7.01-6.73 (m, 4H), 3.86 (s, 2H), 1.88-1.73 (m, 2H), 1.47 (dq, J = 14.7, 7.4 Hz, 2H), 0.96 (t, J = 7.4 Hz, 3H). ^{13}C NMR (100 MHz, $CDCl_3$): δ 144.97, 127.27, 124.38, 122.28, 115.07, 48.38, 28.83, 19.82,13.01.

Synthesis of 2b. A procedure similar to the synthesis of **2a.** The amount of the same substance **1a** was replaced by **1b**, and give 2.52 g (58.0%) of **2b** as a brown oil. ^{1}H NMR (400 MHz, $CDCl_3$): δ 7.25 (s, 1H), 7.25-7.20 (m, 1H), 7.19-7.07 (m, 2H), 6.98-6.82 (m, 2H), 6.69 (dd, J = 7.5, 1.5 Hz, 1H), 4.02-3.61 (m, 2H), 1.91-1.65 (m, 2H), 1.55-1.32 (m, 2H), 1.02-0.78 (m, 3H). ^{13}C NMR (100 MHz, $CDCl_3$): δ 144.98, 144.52, 129.85, 129.60, 127.51, 127.20, 123.99, 122.68, 121.70, 116.58, 115.66, 48.09, 27.95, 19.95, 14.03.

Synthesis of 3b. A procedure similar to the synthesis of **3a.** The amount of the same substance **2a** was replaced by **2b**, and give 1.76 g (48.0%) of **3b** as a white solid. ^{1}H NMR (400 MHz, $CDCl_3$) :δ 8.20 (dd, J = 6.0, 2.4 Hz, 1H), 8.10 (dd, J = 7.9, 1.6 Hz, 1H), 7.66 (dddd, J = 10.3, 8.8, 7.1, 2.0 Hz, 2H), 7.39-7.27 (m, 2H), 7.22 (d, J = 9.1 Hz, 1H), 4.14 (dd, J = 8.8, 6.9 Hz, 2H), 2.00-1.74 (m, 2H), 1.49 (dq, J = 14.9, 7.5 Hz, 2H), 1.07 - 0.94 (m, 3H). ^{13}C NMR (100 MHz, $CDCl_3$): δ 140.64, 139.82, 136.21, 135.93, 133.39, 126.19, 125.49, 123.78, 122.16, 117.84, 116.09, 114.00, 48.30, 28.74, 19.91, 13.72.

Synthesis of CBPDO. A procedure similar to the synthesis of **CEPDO**. The amount of the same substance **3a** was replaced by **3b**, and give 0.27 g (51.2%) of **CBPDO** (m.p. 161.2–162.1 °C) as a white solid. ^{1}H NMR (400 MHz, $CDCl_3$): δ 8.36 (dd, J = 17.3, 2.5 Hz, 1H), 8.19 - 8.10 (m, 3H), 7.81 (dd, J = 9.0, 2.5 Hz, 1H), 7.69 (ddd, J = 8.8, 5.5, 1.6 Hz, 1H), 7.59 (dd, J = 8.7, 5.4 Hz, 1H),

7.47 - 7.28 (m, 8H), 4.32 - 4.23 (m, 2H), 2.02 (dt, J = 15.5, 5.1 Hz, 2H), 1.25 (s, 2H), 1.11 - 0.99 (m, 3H); ^{13}C NMR(100 MHz, $CDCl_3$): δ 140.79, 139.85, 133.39, 131.82, 126.21, 125.34, 124.18, 123.67, 123.49-123.43, 122.16, 120.40, 117.73, 116.14, 109.50, 48.48, 28.93, 20.02, 13.76. Anal. Calcd for $C_{28}H_{24}N_2O_2S$: C, 74.32; H, 5.34; N, 6.19; S, 7.08. Found: C, 74.28; H, 5.35; N, 6.22; S, 7.06.

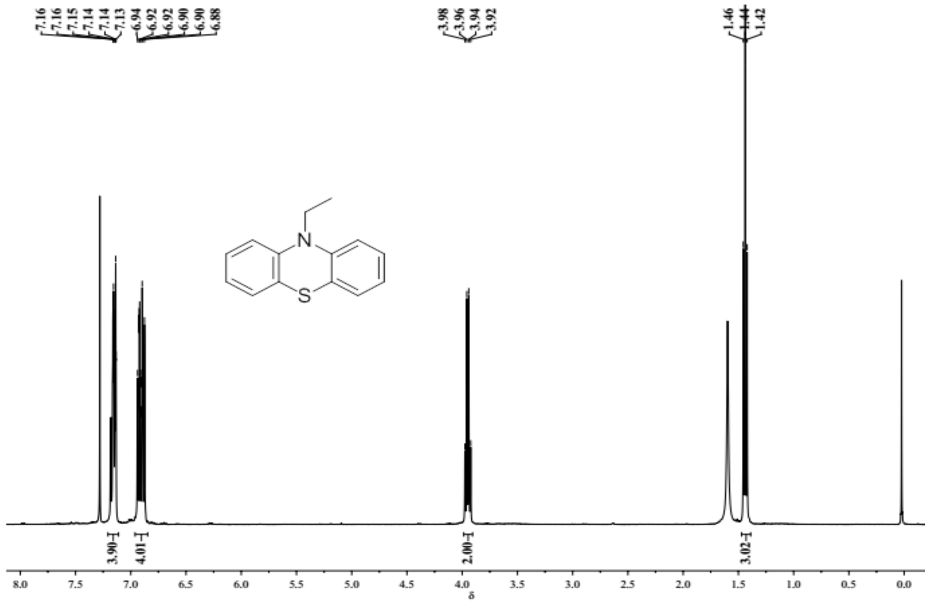

Figure S1: ^{1}H NMR spectrum of organic compound (**1a**).

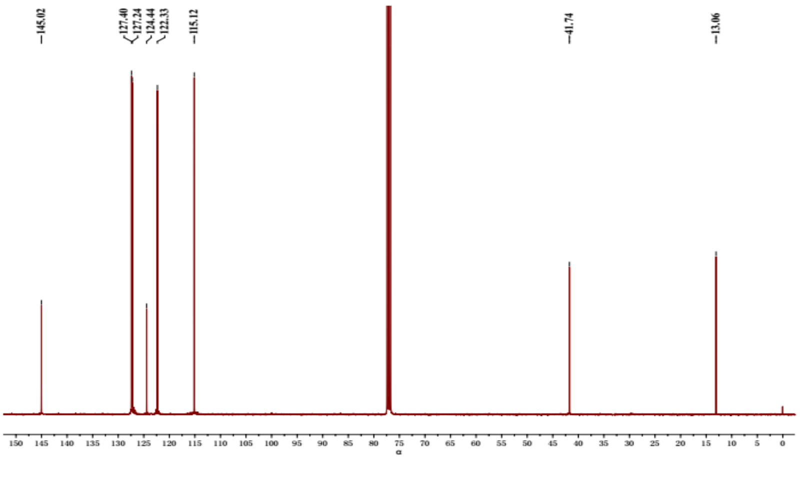

Figure S2: ^{13}C NMR spectrum of organic compound (**1a**).

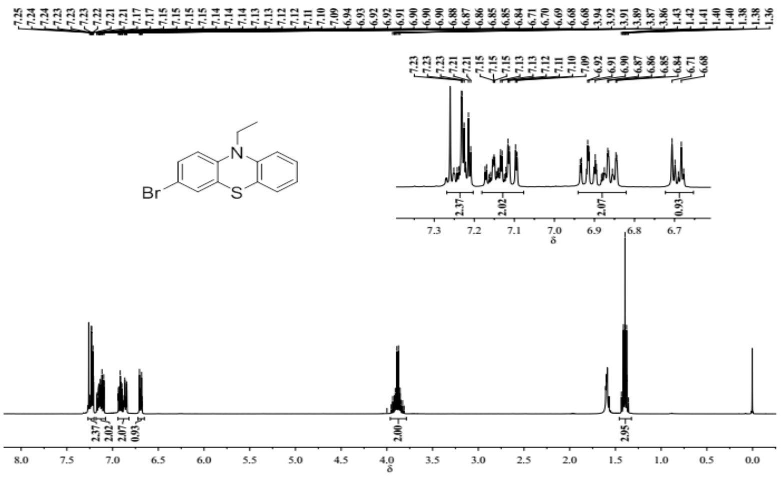

Figure S3: ^{1}H NMR spectrum of organic compound (**2a**).

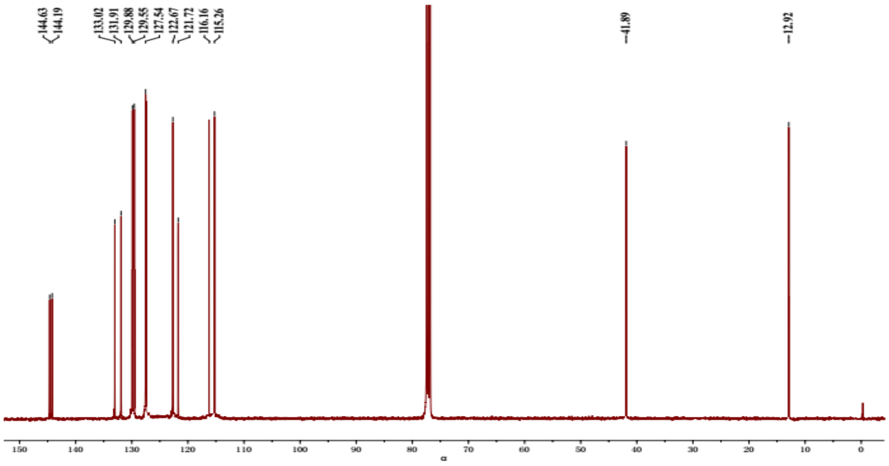

Figure S4:^{13}C NMR spectrum of organic compound (**2a**).

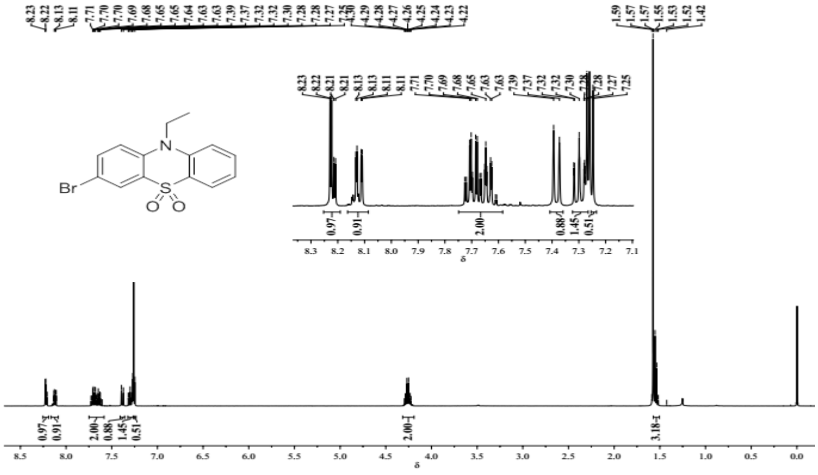

Figure S5: ^{1}H NMR spectrum of compound(**3a**).

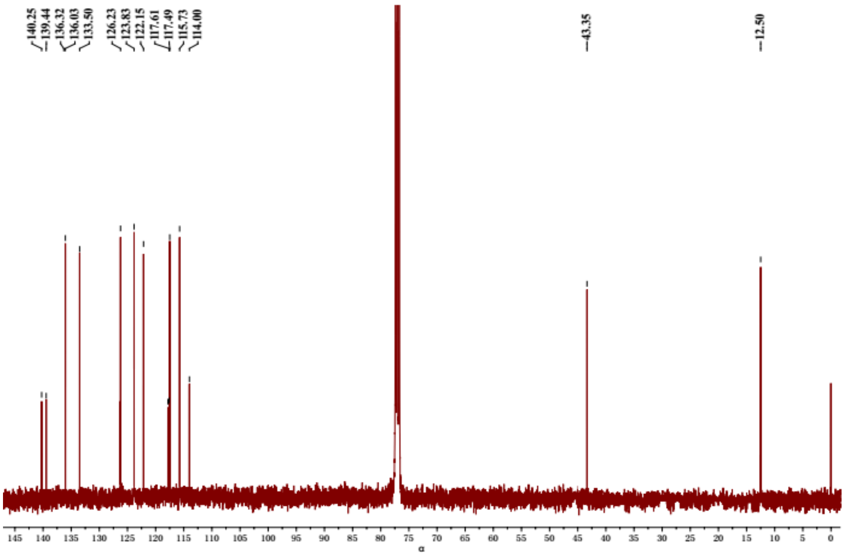

Figure S6: ^{13}C NMR spectrum of compound(**3a**).

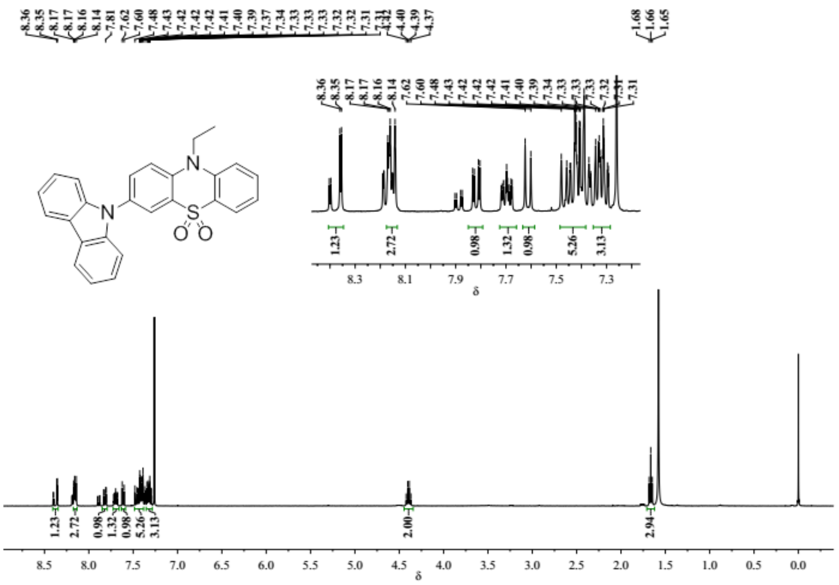

Figure S7: ^{1}H NMR spectrum of compound (CEPDO).

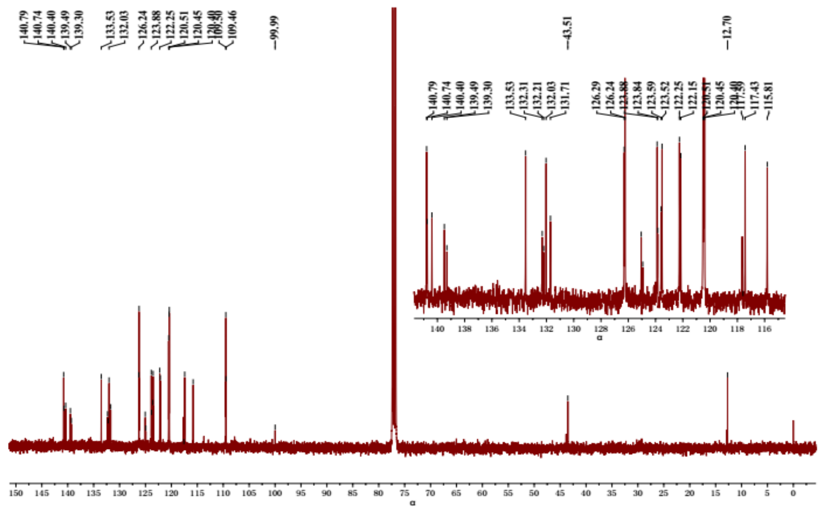

Figure S8. ^{13}C NMR spectrum of compound (CEPDO).

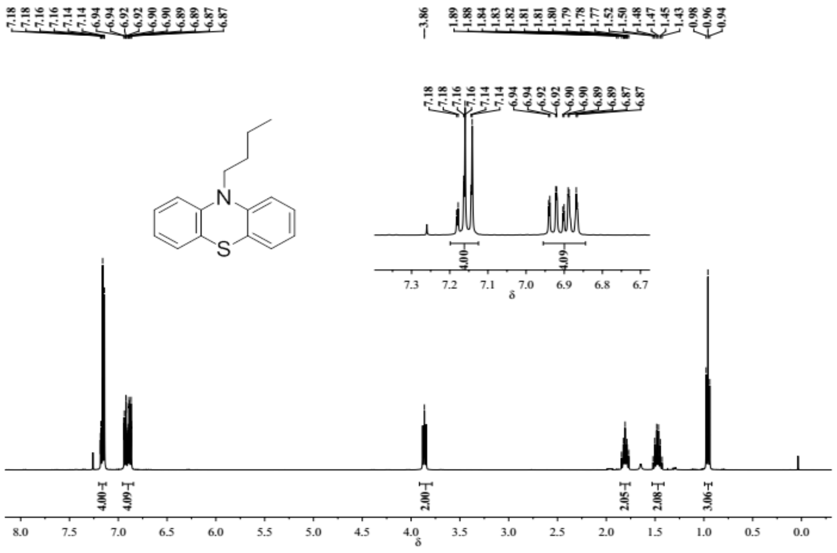

Figure S9: ^{1}H NMR spectrum of compound (**1b**).

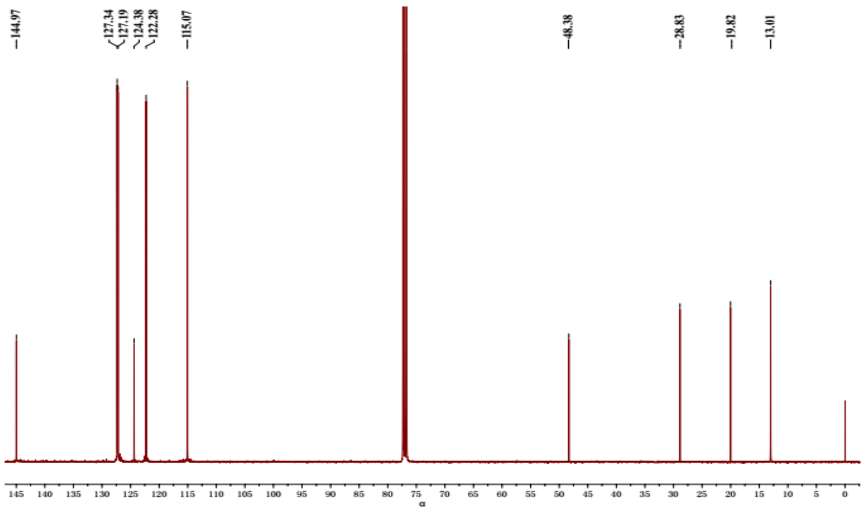

Figure S10: ^{13}C NMR spectrum of compound (**1b**).

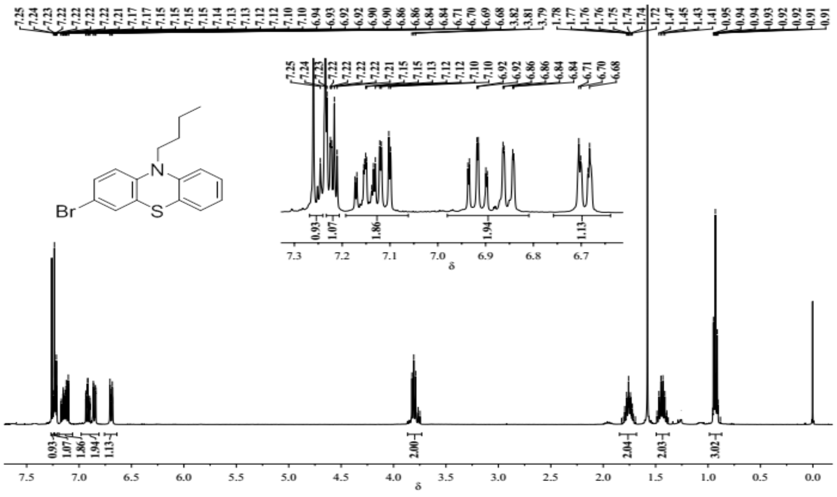

Figure S11: ^{1}H NMR spectrum of compound (**2b**).

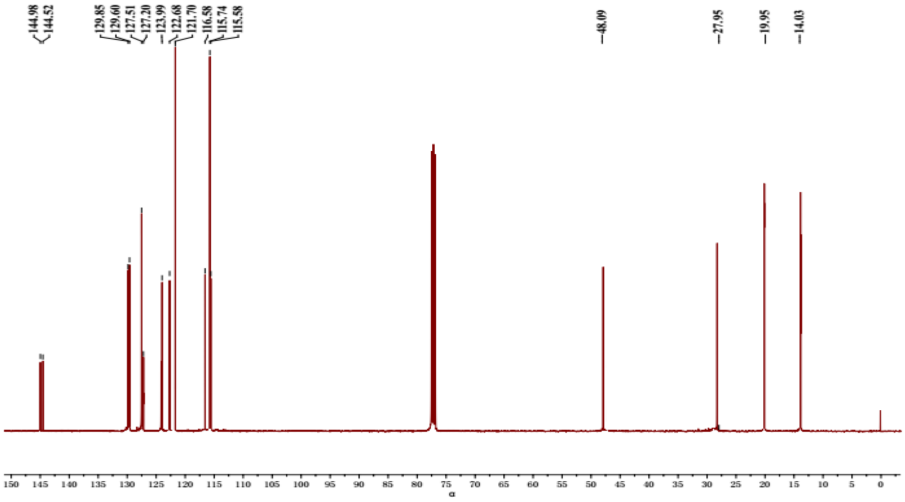

Figure S12: ^{1}H NMR spectrum of compound (**2b**).

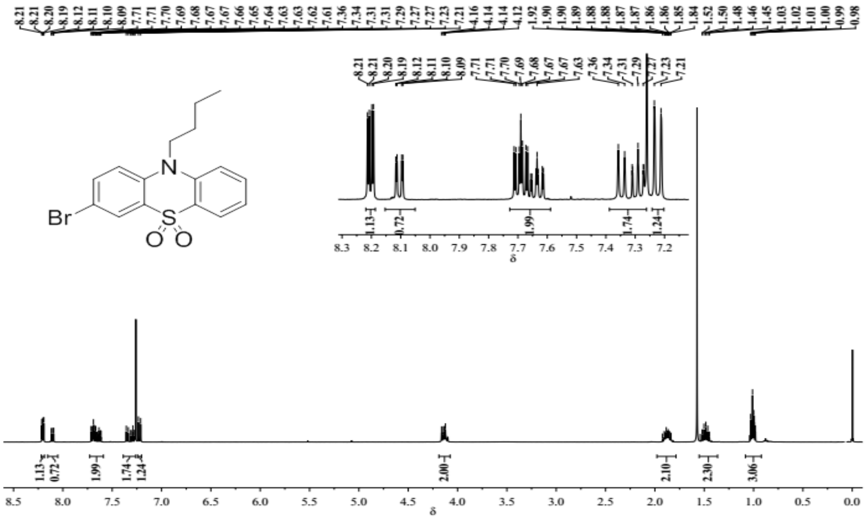

Figure S13: ^{1}H NMR spectrum of compound (**3b**).

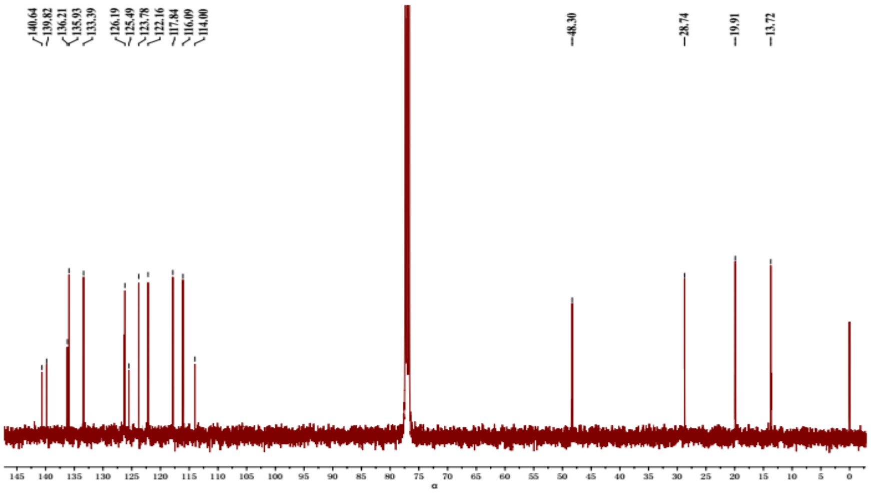

Figure S14: ^{13}C NMR spectrum of compound (**3b**).

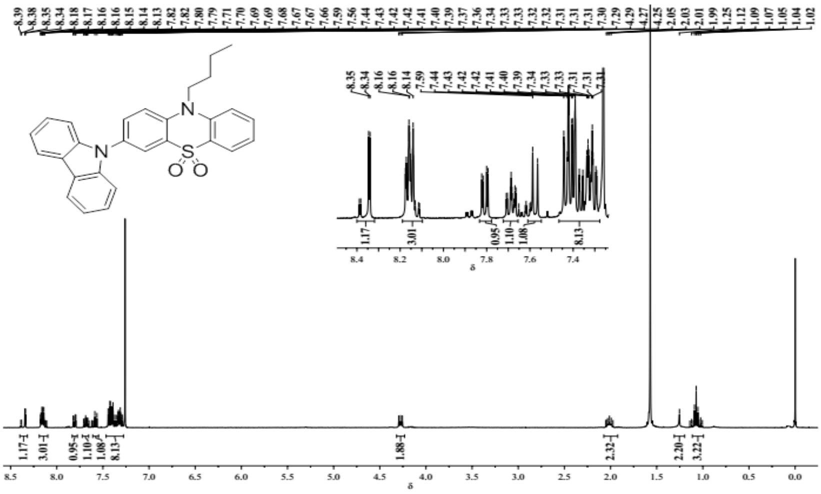

Figure S15: ^{1}H NMR spectrum of compound (CBPDO).

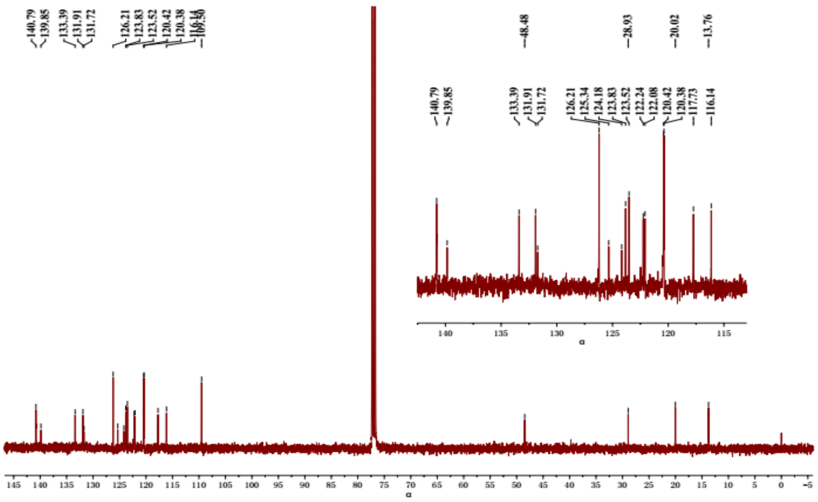

Figure S16: ^{13}C NMR spectrum of compound (CBPDO).

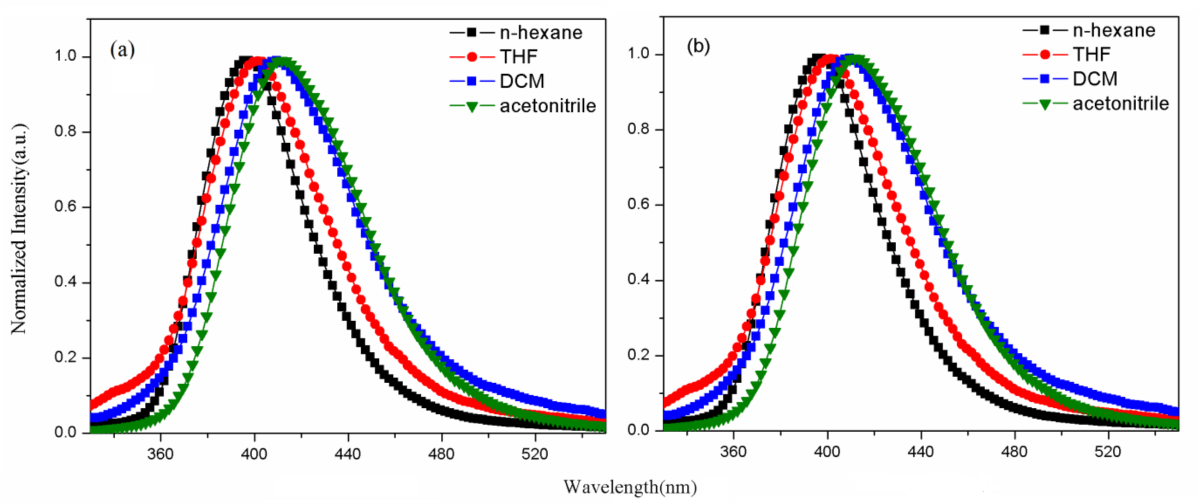

Figure S17: PL spectra of (a) CEPDO and (b) CBPDO dissolved in different solvents.
